# KLF15 Is a Molecular Link between Endoplasmic Reticulum Stress and Insulin Resistance

**DOI:** 10.1371/journal.pone.0077851

**Published:** 2013-10-22

**Authors:** Dae Young Jung, UmaDevi Chalasani, Ning Pan, Randall H. Friedline, Domenick A. Prosdocimo, Minwoo Nam, Yoshihiro Azuma, Rajanikanth Maganti, Kristine Yu, Ashish Velagapudi, Bryan O’Sullivan-Murphy, Juliano L. Sartoretto, Mukesh K. Jain, Marcus P. Cooper, Fumihiko Urano, Jason K. Kim, Susan Gray

**Affiliations:** 1 Program in Molecular Medicine, University of Massachusetts Medical School, Worcester, Massachusetts, United States of America; 2 Division of Endocrinology, Metabolism and Diabetes, University of Massachusetts Medical School, Worcester, Massachusetts, United States of America; 3 Division of Cardiovascular Medicine, University of Massachusetts Medical School, Worcester, Massachusetts, United States of America; 4 Department of Medicine, University of Massachusetts Medical School, Worcester, Massachusetts, United States of America; 5 Department of Medicine, Division of Endocrinology, Metabolism, and Lipid Research, Washington University School of Medicine, St. Louis, Missouri, United States of America; 6 Department of Medicine, Case Western Reserve University School of Medicine, Cleveland, Ohio, United States of America; 7 Cardiovascular Division, Brigham and Women's Hospital, Harvard Medical School, Boston, Massachusetts, United States of America; University of Texas Health Science Center at San Antonio, United States of America

## Abstract

Obesity places major demands on the protein folding capacity of the endoplasmic reticulum (ER), resulting in ER stress, a condition that promotes hepatic insulin resistance and steatosis. Here we identify the transcription factor, Kruppel-like factor 15 (KLF15), as an essential mediator of ER stress-induced insulin resistance in the liver. Mice with a targeted deletion of *KLF15* exhibit increased hepatic ER stress, inflammation, and JNK activation compared to WT mice; however, *KLF15* ^*-/-*^ mice are protected against hepatic insulin resistance and fatty liver under high-fat feeding conditions and in response to pharmacological induction of ER stress. The mammalian target of rapamycin complex 1 (mTORC1), a key regulator of cellular energy homeostasis, has been shown to cooperate with ER stress signaling pathways to promote hepatic insulin resistance and lipid accumulation. We find that the uncoupling of ER stress and insulin resistance in *KLF15* ^*-/-*^ liver is associated with the maintenance of a low energy state characterized by decreased mTORC1 activity, increased AMPK phosphorylation and PGC-1α expression and activation of autophagy, an intracellular degradation process that enhances hepatic insulin sensitivity. Furthermore, in primary hepatocytes, KLF15 deficiency markedly inhibits activation of mTORC1 by amino acids and insulin, suggesting a mechanism by which KLF15 controls mTORC1-mediated insulin resistance. This study establishes KLF15 as an important molecular link between ER stress and insulin action.

## Introduction

A variety of stimuli, including changes in nutrient availability, oxidative stress, pathogen infection and abnormal Ca^2+^ regulation, can result in endoplasmic reticulum (ER) stress, characterized by the accumulation of misfolded proteins in the ER lumen (reviewed in 1). In order to adapt to ER stress, multiple signaling pathways are activated as part of the unfolded protein response (UPR). To date, three main ER transmembrane proteins have been identified that serve as ER stress sensors and activators of the UPR: inositol-requiring kinase 1α (IRE1α), activating transcription factor 6 (ATF6) and double-stranded RNA-dependent protein kinase (PKR)-like ER kinase (PERK). Under normal physiological conditions, association of these proteins with the ER lumen chaperone, 78 kD glucose-regulated protein (GRP78), represses their activity. ER stress promotes the dissociation of GRP78, permitting UPR signaling to occur [2], [3]. Once activated, IRE1α and ATF6 counteract ER stress primarily via the induction of genes encoding proteins that facilitate protein folding or degradation [4], [5], while PERK functions mainly to decrease protein translation by phosphorylating the alpha subunit of eukaryotic translation initiation factor 2 (eIF2α) [6]. 

ER stress plays a critical role in the development of hepatic insulin resistance (reviewed in 7). Hotamisligil and colleagues [8] were the first to show that ER stress is increased in both dietary and genetic models of mouse obesity. They further found that ER stress reduces the activity of the insulin signaling pathway via hyperactivation of c-Jun N-terminal kinase (JNK). Multiple mechanisms have been shown to underlie the activation of JNK in response to ER stress, including the recruitment of JNK by an IRE1α-TNF receptor-associated factor 2 (TRAF2) complex [9], calcium release from the ER and mitochondrial production of reactive oxygen species (ROS) [10], [11]. Activated JNK can promote insulin resistance through serine phosphorylation of insulin receptor substrate 1 (IRS-1) and also via the induction of proinflammatory cytokines that feed back and further activate JNK [12]. ER stress also induces nuclear factor kappa B (NFΚB) signaling, which, like JNK signaling, can promote insulin resistance via the induction of inflammatory genes [13].

Recent studies have revealed the importance of autophagy, a lysosomal pathway-mediated degradation process, in the regulation of ER stress-induced insulin resistance [14]. During autophagy, cytoplasmic components ranging from protein aggregates to whole organelles are sequestered into a double-membrane vesicle that fuses with the lysosome, where vesicle contents are degraded (reviewed in 15). ER stress has been shown to activate autophagy [16], and like the UPR, autophagy may help to restore ER homeostasis [17]. In genetic and diet-induced obesity, autophagy is markedly downregulated in the liver, leading to reduced hepatic insulin action and increased ER stress, and restoration of autophagy increases insulin sensitivity [14], [18]. Mammalian target of rapamycin complex 1 (mTORC1), a central regulator of protein synthesis and cell growth, is a key inhibitor of autophagy in response to growth factors and nutrients [19] and is aberrantly hyperactivated in the obese state [20]. Thus, mTORC1-mediated inhibition of autophagy may play a key role in promoting insulin resistance. 

Our current findings identify KLF15 as a critical regulator of ER stress and autophagy signaling as well as an effector of insulin and nutrient-mediated activation of mTORC1. Kruppel-like factor 15 (KLF15) is a zinc finger DNA-binding protein belonging to the Kruppel-like family of transcription factors [21], [22] and a key regulator of metabolic pathways that control adipogenesis [23], gluconeogenesis [24], [25], circadian nitrogen homeostasis [26] and exercise adaptation in skeletal muscle [27]. We now show that KLF15 plays an essential role in ER stress-mediated insulin resistance in the liver.

## Results

### Metabolic responses of KLF15-deficient mice to high-fat feeding

**Figure 1:**
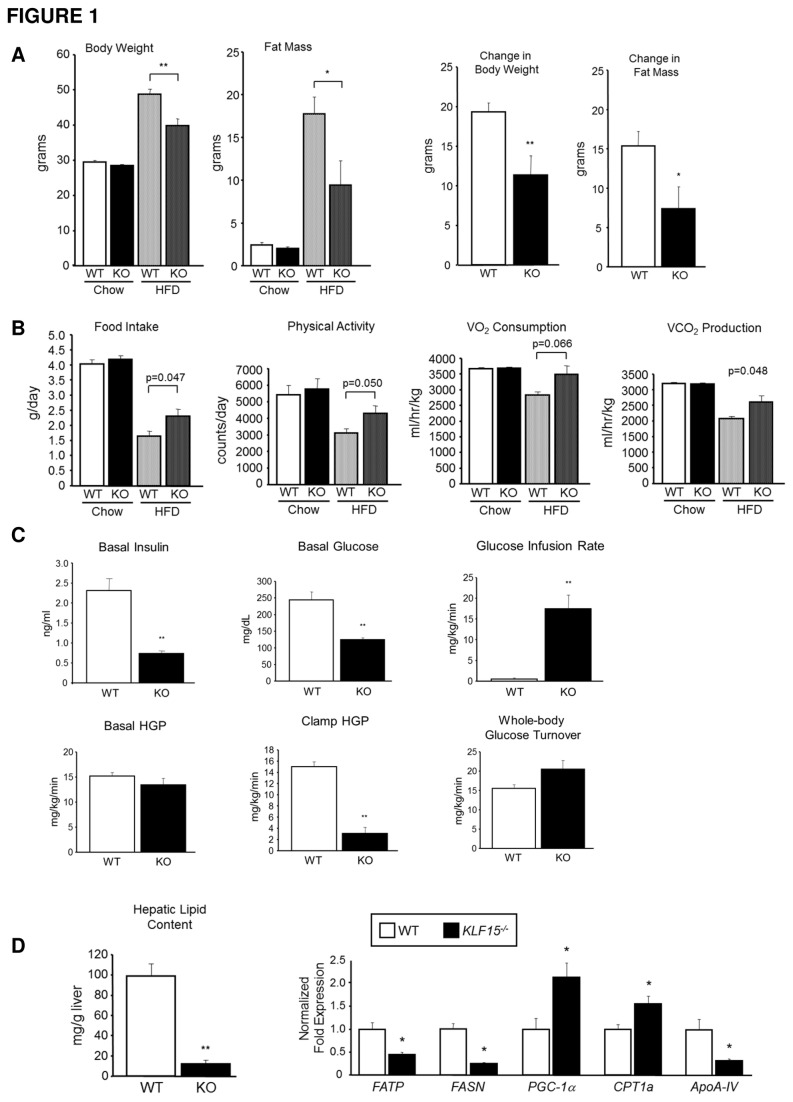
Metabolic responses to high-fat feeding in KLF15-deficient versus control mice. Male mice were placed on a high-fat diet (HFD; 60% kcal from fat) at 3-4 months of age. **A**. **Body composition**. Body weight (n=11-12) and whole-body fat mass (n=5-6) in wild-type (WT) and *KLF15* ^*-/-*^ (KO) mice were measured immediately prior to (chow) and 13 weeks after the start of HFD. Right panels indicate change in body weight and fat mass during 13 week HFD period. **B**. **Metabolic cage analysis**. Assessment of food intake, physical activity, O_2_ consumption and CO_2_ production immediately prior to (chow) and 12 weeks after the start of HFD (n=6). **C**. **Hyperinsulinemic-euglycemic clamp study**. Clamp procedure was performed after 14 weeks of HFD. Top (left to right): basal plasma insulin levels after an overnight fast, basal plasma glucose levels after an overnight fast, glucose infusion rate during the insulin clamp. Bottom (left to right): basal hepatic glucose production (HGP) after an overnight fast (prior to insulin stimulation), hepatic glucose production during the insulin clamp, and insulin-stimulated whole-body glucose turnover (peripheral glucose uptake; n=5; results are representative of two individual experiments). **D**. **Hepatic lipid content and lipid-related gene expression in WT versus *KLF15*^*-/-*^ mice**. After 14 weeks of HFD, liver tissue from clamped mice was collected and flash frozen. To assess hepatic lipid content, a triglyceride assay was performed using a commercially available kit (left; n=5-7). To measure lipid-related gene expression, total RNA was isolated from liver tissue, reverse transcribed and amplified by QPCR using primers against the genes shown (right; n=3). Figure 1 statistical comparisons were made using Student’s t test for unpaired samples. Values = mean ± SEM; *p<0.05; **p<0.01 compared to WT control.

To evaluate the metabolic effects of diet-induced obesity in *KLF15* ^*-/-*^ mice, we placed 3-4 month-old male WT and *KLF15* ^*-/-*^ mice on a high-fat diet (HFD; 60% kcal from fat) for 14 weeks. As shown in Figure 1A, *KLF15* ^*-/-*^ mice gain significantly less body weight and fat mass than WT controls after 13 weeks of HFD. Metabolic cage analyses (Figure 1B) showed no significant differences between standard chow-fed WT and *KLF15* ^*-/-*^ mice in food intake, physical activity or energy expenditure (VO_2_ consumption and VCO_2_ production). After 12 weeks of HFD, daily food intake is significantly increased in *KLF15* ^*-/-*^ versus WT mice; however, physical activity level and energy expenditure are also increased in these mice, consistent with decreased whole-body fat mass compared to WT controls. 

To assess whole-body insulin sensitivity, intraperitoneal glucose tolerance tests (GTT) were performed after 8 weeks of high-fat feeding (Figure S1). *KLF15* ^*-/-*^ mice show markedly increased glucose tolerance compared to WT mice as indicated by significantly reduced area under the curve of glucose clearance. Plasma insulin levels during the GTT indicate that improved glucose clearance in *KLF15* ^*-/-*^ versus WT controls is not due to increased insulin secretion. After 14 weeks of HFD, organ-specific insulin sensitivity was measured using hyperinsulinemic-euglycemic clamp analysis on conscious WT and *KLF15* ^*-/-*^ mice (Figure 1C). Immediately prior to the clamp, plasma was collected from overnight-fasted mice and assayed for insulin and glucose concentrations. Consistent with their insulin-sensitive phenotype, *KLF15* ^*-/-*^ mice showed a significant 3-fold decrease in basal insulin levels versus WT controls. Basal glucose levels in *KLF15* ^*-/-*^ mice were also significantly decreased compared to WT controls. During the clamp, *KLF15* ^*-/-*^ mice were more insulin sensitive than WT mice, requiring a greater than 30-fold increase in glucose infusion rate to maintain euglycemia. Although basal hepatic glucose production (HGP) was similar between groups, we found a substantial and statistically significant decrease in HGP under insulin clamp conditions in *KLF15* ^*-/-*^ mice versus WT controls. A trend of increased whole body glucose turnover (peripheral glucose uptake) was observed in *KLF15* ^*-/-*^ mice that did not reach statistical significance. Thus, under high-fat feeding conditions, *KLF15* ^*-/-*^ mice are more insulin sensitive than WT controls, primarily as a result of decreased hepatic glucose production. We cannot rule out the possibility that different results may be achieved by changing the age at which HFD is introduced or the duration of high-fat feeding; however, our data indicate that in mature mice, long-term high-fat feeding leads to significantly increased insulin resistance in WT versus *KLF15* ^*-/-*^ liver. 

The association of hepatic insulin resistance with increased hepatic lipid content is well-documented, and the mechanisms underlying this association are the focus of current investigation (reviewed in 28). As shown in Figure 1D (left), WT mice accumulate ~10-fold more hepatic lipid than *KLF15* ^*-/-*^ mice after 14 weeks of high-fat feeding. Thus, *KLF15* ^*-/-*^ mice are protected against HFD-induced hepatic steatosis. mRNA expression of lipid-related genes in these tissues was assessed using real-time quantitative PCR (QPCR) analysis (Figure 1D, right). Consistent with decreased hepatic lipid content, *KLF15* ^*-/-*^ liver exhibited reduced mRNA levels of fatty acid transport protein (FATP), fatty acid synthase (*FASN*) and Apolipoprotein A-IV (*ApoA-IV*), which are involved in fatty acid uptake/synthesis and triglyceride secretion, respectively. Increased hepatic expression of peroxisome proliferator-activated receptor gamma coactivator-1 alpha (*PGC-1α*) and carnitine palmitoyl transferase 1a (*CPT 1a*) in *KLF15* ^*-/-*^ versus WT mice suggests that enhanced fatty acid oxidation (FAO) may play a role in reducing hepatic lipid levels in *KLF15* ^*-/-*^ mice. Additional evidence for increased hepatic FAO in these mice comes from the observation of elevated fasting concentrations of the ketone, β-hydroxybutyrate, in whole blood from HFD-fed *KLF15* ^*-/-*^ versus WT mice (Figure S2). Thus, the absence of KLF15 inhibits the accumulation of hepatic lipid in response to high-fat feeding, potentially via a mechanism involving increased fatty acid oxidation.

### KLF15 regulates the ER stress response

**Figure 2:**
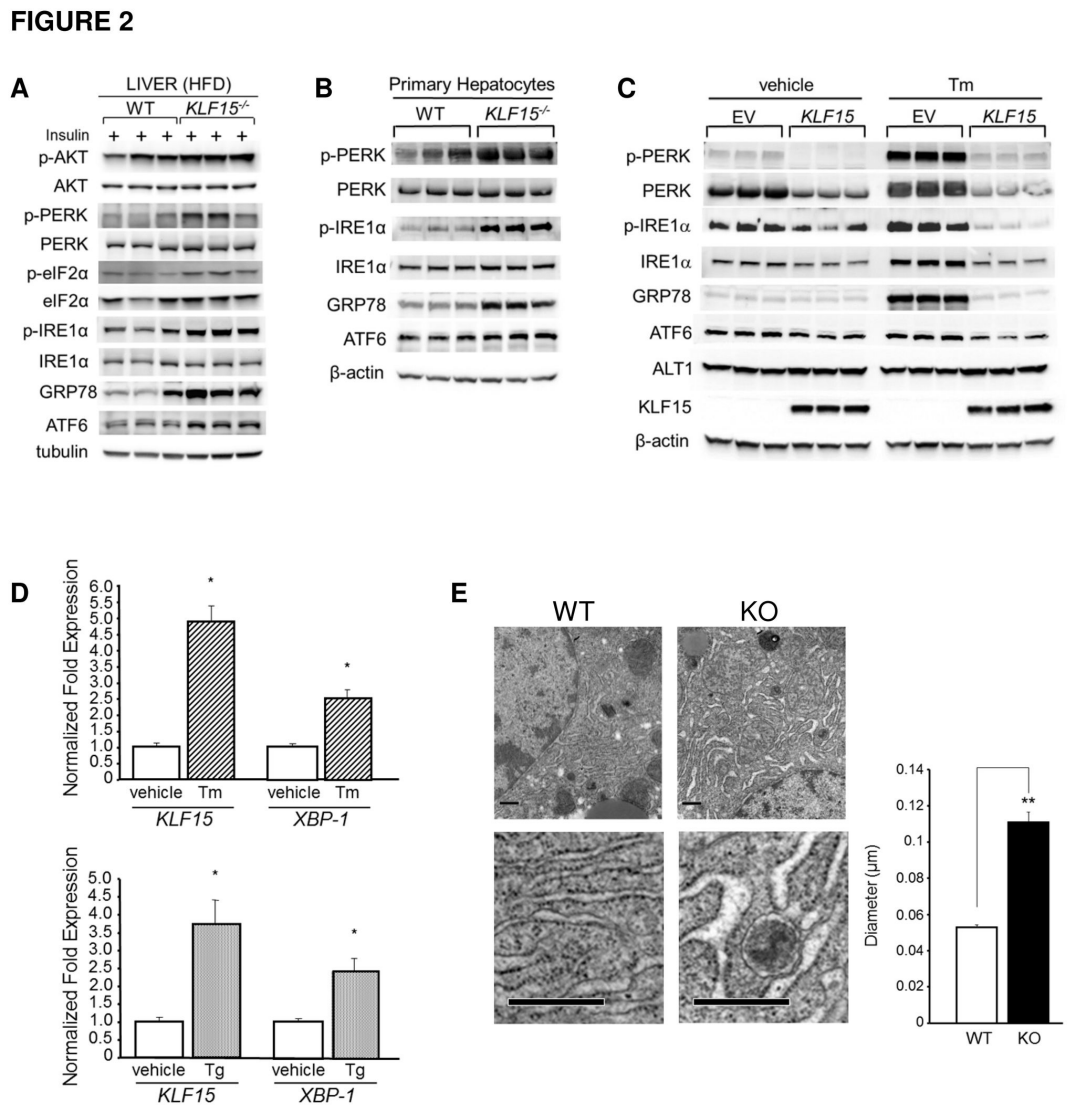
Regulation of the unfolded protein response by KLF15. **A**. **UPR activity in WT versus *KLF15*^*-/-*^ liver after high-fat feeding**. 2-month-old WT and *KLF15* ^*-/-*^ male mice received HFD (60% kcal from fat) for 8 weeks, then were fasted for 5h, i.p. injected with 10mU/g insulin and sacrificed 10 minutes post-injection. Liver lysates were subjected to immunoblotting (n=3). **B**. **Western analysis of UPR activity in WT versus *KLF15*^*-/-*^ primary hepatocytes**. Hepatocytes were isolated from standard chow-fed 4-month-old male WT and *KLF15* ^*-/-*^ mice. **C**. **Effect of KLF15 overexpression on UPR activity**. Western analysis of hepatocytes isolated from chow-fed 4-month-old WT male mice infected with empty vector (EV) or *KLF15*-expressing adenovirus. Cells were treated for 6h with vehicle (DMSO) or 5µg/ml tunicamycin (Tm) before harvest. **D**. **Induction of KLF15 in response to acute ER stress**. AML-12 hepatocytes were treated for 7h hours with vehicle or 5µg/ml tunicamycin (Tm; top panel) or for 5h with vehicle or 1µM thapsigargin (Tg; bottom panel). Total RNA was isolated from harvested cells, reverse transcribed and subjected to QPCR. **E**. **Ultrastructural examination of ER morphology**. WT and *KLF15* ^*-/-*^ female mice (n=3) received HFD (60% kcal from fat) for 4 weeks starting at age 3 months. Mice were fasted for 21h before sacrifice and liver tissue was removed and prepared for transmission electron microscopy analysis as described in Materials and Methods. Left: Representative electron micrographs (original magnification = 20,500x) of liver sections. Lower pictures show an enlarged portion of the field above. Scale bar = 0.5 μm. Right: Quantitation of ER lumen diameter. Values = average lumen diameter/cell representative of 9 cells/group. In each cell, lumen diameter measurements were taken along the length of each of 10 ER cisternae. For Figure 2B, C and D, two individual experiments were performed in triplicate; each lane (in B, C) indicates a technical replicate. For Figure 2A, each lane represents one mouse. Values = mean ± SEM; *p<0.05; **p<0.01 (Student’s t test for unpaired samples).

Consistent with the clamp data, phosphorylation of the serine/threonine kinase, AKT, is increased in livers isolated from insulin-stimulated *KLF15* ^*-/-*^ versus WT mice after 8 weeks of HFD; however, these mice also exhibit increased hepatic expression and phosphorylation of UPR genes compared to WT littermates, providing evidence for uncoupling of ER stress and insulin resistance (Figure 2A). We also find that UPR factor expression and activity is significantly increased in hepatocytes isolated from *KLF15* ^*-/-*^ versus WT mice, suggesting a liver autonomous effect of KLF15 on ER stress signaling (Figure 2B). To further investigate the regulation of the UPR by KLF15, we introduced exogenous KLF15 into WT primary hepatocytes via adenoviral infection and treated infected cells with vehicle or with tunicamycin (Tm; an inhibitor of N-linked glycosylation) to induce acute ER stress. As shown in Figure 2C, overexpression of KLF15 suppresses both basal and Tm-induced expression and phosphorylation (where applicable) of the critical UPR regulators PERK, IRE1α, ATF6 (full-length) and GRP78. The induction of the amino acid degrading enzyme alanine aminotransferase (ALT1), a known target of KLF15, is included as a positive control. We also assessed the effect of acute ER stress on KLF15 expression. Figure 2D shows that in an AML-12 hepatocyte cell line, *KLF15* mRNA levels increase several-fold in response to treatment with Tm or another common inducer of ER stress, thapsigargin (Tg; a Ca^2+^-ATPase inhibitor). X-box-binding protein 1 (*XBP-1*) expression levels are included as a positive control. Thus, like UPR factors, KLF15 may be induced with ER stress in order to restore ER homeostasis. 

Elevated UPR activity in KLF15-deficient mice suggests a chronic increase in ER stress; however, it may simply be an indication that KLF15 is an upstream regulator of the UPR. To distinguish between these possibilities, we performed ultrastructural examination of ER morphology in liver tissue isolated from HFD-fed WT and *KLF15* ^*-/-*^ mice using transmission electron microscopy. As shown in Figure 2E, the rough ER appears markedly dilated in *KLF15* ^*-/-*^ compared with WT hepatocytes, with a significant increase in average diameter of the ER lumen. This indicates that the absence of KLF15 causes ER stress, and that increased UPR signaling in *KLF15* ^*-/-*^ liver occurs, at least in part, as a response to this condition. At this time, it is unclear whether KLF15 may also affect ER stress signaling as a direct transcriptional regulator of UPR gene expression. For example, we find that overexpression of KLF15 in WT hepatocytes significantly reduces transcript levels of *GRP78* and *PERK*; however, preliminary chromatin immunoprecipitation analyses have not yet identified definitive KLF15 binding sites in the proximal promoter regions of these genes (data not shown).

**Figure 3:**
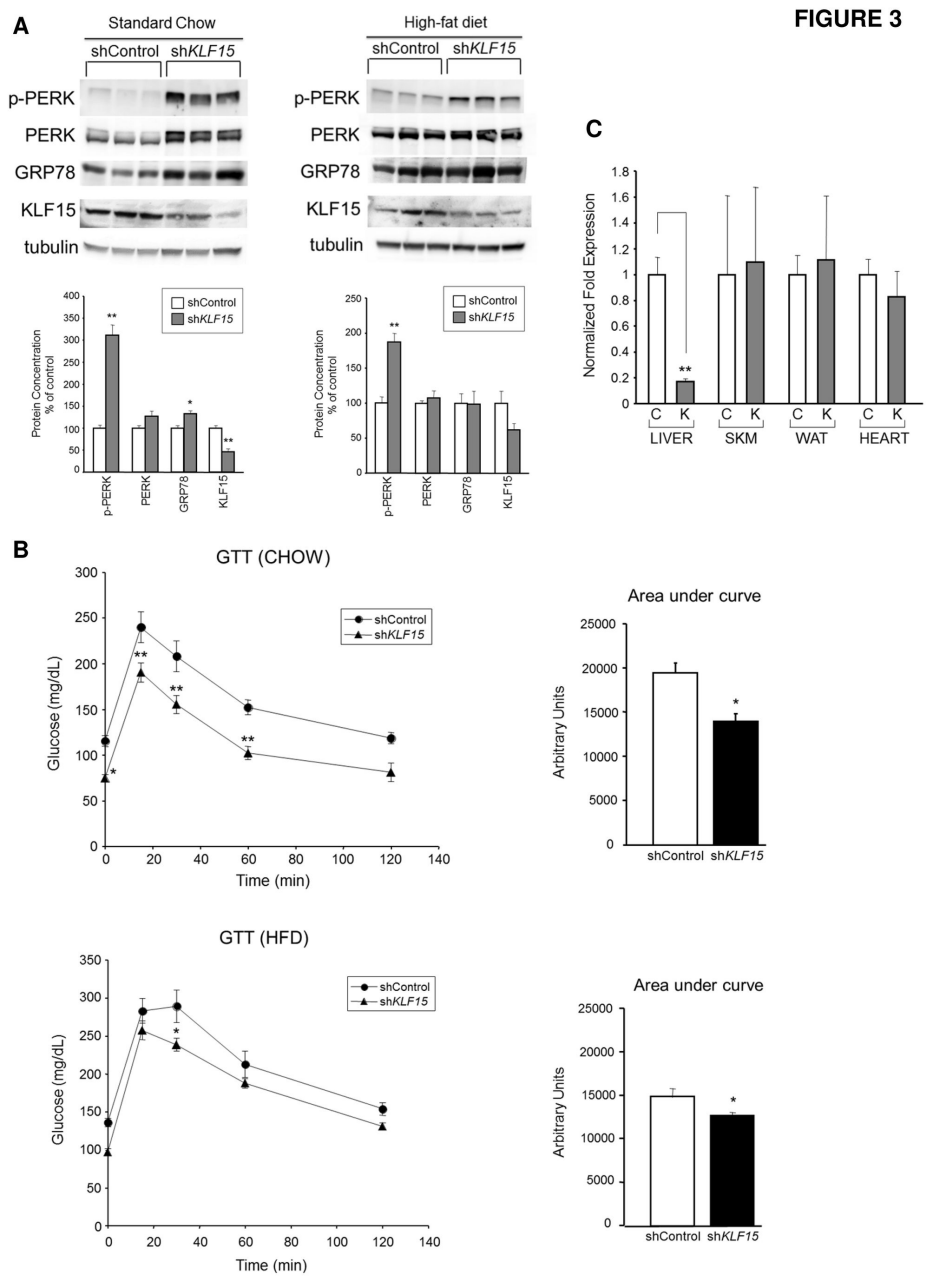
Uncoupling of ER stress and insulin resistance in mice with a liver-specific knockdown of KLF15. **A**. **Effect of liver-specific knockdown of KLF15 on the hepatic UPR**. C57BL/6 male mice received a tail vein injection of 1.5 x 10^11^ viral particles of either shControl or sh*KLF15* adenovirus under chow feeding conditions at 13 weeks of age or after 5 weeks of high-fat feeding at 14 weeks of age. Chow-fed mice (Day 4 post-injection) and HFD-fed mice (Day 6 post-injection) were fasted for 4h followed by i.p. injection of 10mU/g insulin and were sacrificed 10 minutes post-insulin injection. Liver lysates were subjected to Western analysis with the indicated antibodies. Each lane represents one mouse (n=3). Quantitation graphs are shown below blots. **B**. **Glucose tolerance tests**. C57BL/6 male mice received a tail vein injection of 1.5 x 10^11^ viral particles of shControl or sh*KLF15* adenovirus under chow feeding conditions at 10 weeks of age or after 5 weeks of high-fat feeding at 14 weeks of age. Four days after tail vein injection, mice were injected i.p. with 1g glucose/kg body weight after a 16h fast. Tail vein blood samples were assessed for glucose concentration immediately before i.p. injection (Time 0) and at 15, 30, 60 and 120 minutes post-injection. Blood glucose concentrations during the GTT and corresponding area under the curve calculations for glucose values are shown for chow- and HFD-fed mice. n=6-7 mice/group for both studies. **C**. **Liver specificity of KLF15 knockdown**. Quantitative PCR evaluation of *KLF15* mRNA levels in liver, quadriceps muscle, white adipose and heart tissues isolated from chow-fed mice that were tail vein-injected with shControl (C) or sh*KLF15* (K) adenovirus. n=3. Figure 3 statistical comparisons were made using Student’s t test for unpaired samples or, for glucose values during GTT, analysis of variance for repeated measures with a Bonferroni post hoc test. Values = mean ± SEM; *p<0.05; **p<0.01 compared to WT control.

Further support for liver-autonomous effects of KLF15 on ER stress signaling comes from analysis of mice with a liver-specific knockdown of KLF15 generated via tail vein injection of adenoviral small hairpin RNA (shRNA). As shown in Figure 3A, reduction of hepatic KLF15 expression in chow-fed mice significantly increases GRP78 expression and PERK phosphorylation compared to control mice (left panel). A similar trend is seen in HFD-fed mice; however, induction of GRP78 expression in response to KLF15 knockdown does not reach statistical significance (right panel). Furthermore, mice with a liver-specific knockdown of KLF15 show increased whole-body insulin sensitivity compared to WT controls under both chow and high-fat feeding conditions (Figure 3B), supporting a liver-autonomous role for KLF15 in the regulation of glucose homeostasis. QPCR amplification of first-strand cDNA templates from multiple tissue types isolated from chow-fed control and KLF15 knockdown mice provides evidence for liver-specific targeting of *KLF15* (Figure 3C). Our data indicate that uncoupling of ER stress and insulin resistance results from liver autonomous effects of KLF15 deficiency and is independent of dietary conditions. 

### KLF15 mediates ER stress-induced hepatic lipid accumulation and insulin resistance

**Figure 4:**
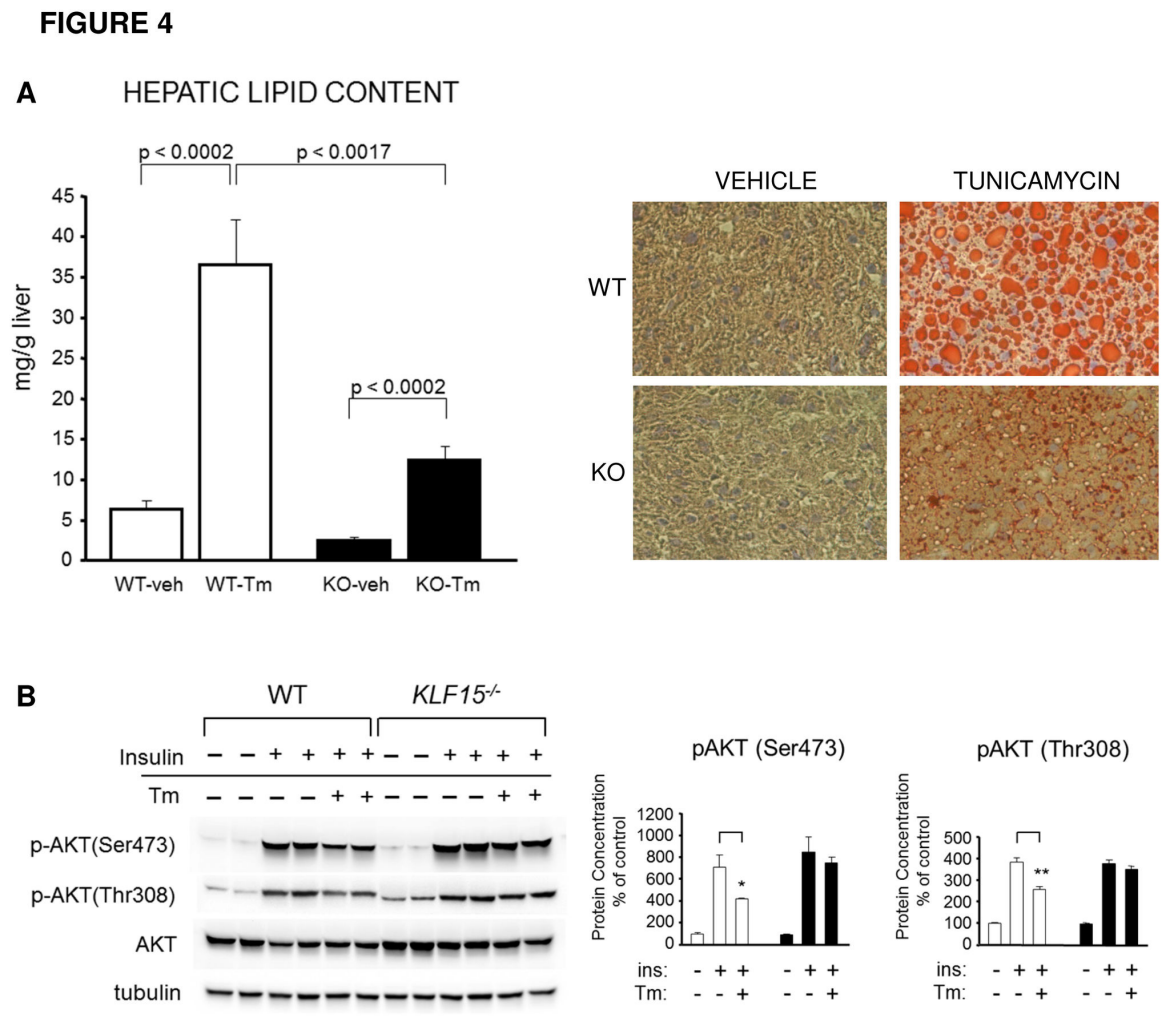
Protection against ER stress-induced hepatic lipid accumulation and insulin resistance *KLF15* ^*-/-*^ liver. **A**. **Hepatic lipid accumulation in response to tunicamycin treatment in WT versus *KLF15*^*-/-*^ mice**. 5-month-old male WT and *KLF15* ^*-/-*^ mice on a standard chow diet with free access to food were i.p. injected with vehicle or 3mg/kg tunicamycin (Tm). 24h post injection, mice were sacrificed and liver tissue removed and flash frozen. Liver tissues were subjected to triglyceride assay with a commercially available kit (left; n=7). Oil Red O staining (right) was performed on frozen liver sections for detection of neutral lipid. Representative samples are pictured. **B**. **Effect of acute ER stress on AKT activity in WT versus *KLF15*^*-/-*^ primary hepatocytes**. Hepatocytes were isolated from standard chow-fed 2.5-month-old male WT and *KLF15* ^*-/-*^ mice and treated for 10 minutes with vehicle, 100nM insulin or 100nM insulin preceded by 20h treatment with 2µg/ml tunicamycin (Tm). Lysates were subjected to immunoblotting with antibodies against total and phospho-AKT (Ser473, Thr308). Three individual experiments were performed in triplicate; each lane indicates a technical replicate. Quantitation graph shown next to immunoblot (white bar = WT, black bar = KLF15^-/-^). Figure 4 statistical analysis was performed using Student’s t-test for unpaired samples. Values = mean ± SEM. *p<0.05.

ER stress plays an important role in the development of fatty liver. Under high-fat feeding conditions, protection against hepatic steatosis in *KLF15* ^*-/-*^ mice is associated with increased hepatic ER stress, suggesting that KLF15 may be required for ER stress-induced hepatic lipid accumulation. To test this hypothesis, chow-fed WT and *KLF15* ^*-/-*^ mice received an intraperitoneal injection of vehicle or 3 mg/kg Tm and livers were taken 24h post-injection. As shown in Figure 4A (left), in response to Tm treatment, hepatic triglyceride content in WT mice increases by ~30mg/g liver versus an ~10mg/g liver increase in *KLF15* ^*-/-*^ mice. These results are supported by Oil Red O staining of frozen liver sections from these mice (Figure 4A, right). Thus, compared to WT controls, *KLF15* ^*-/-*^ mice are protected against ER stress-induced fatty liver. Protection against both HFD and ER stress-induced hepatic steatosis is likely to contribute to increased hepatic insulin sensitivity in *KLF15* ^*-/-*^ mice.

Because *KLF15* ^*-/-*^ mice show indications of chronic ER stress but remain insulin sensitive compared to WT littermates, we hypothesized that KLF15 may mediate ER stress-induced insulin resistance. To test this, we treated primary hepatocytes isolated from chow-fed WT and *KLF15* ^*-/-*^ mice with vehicle, insulin only, or insulin preceded by a 20h treatment with 2µg/ml Tm (Figure 4B). We found that Tm pretreatment significantly decreased insulin-stimulated phosphorylation of AKT in WT, but not in *KLF15* ^*-/-*^ hepatocytes. These results indicate that, under acute ER stress conditions, the absence of KLF15 enhances insulin signaling pathway activity. 

### JNK activation and inflammation are uncoupled from insulin resistance in KLF15^-/-^ liver

**Figure 5:**
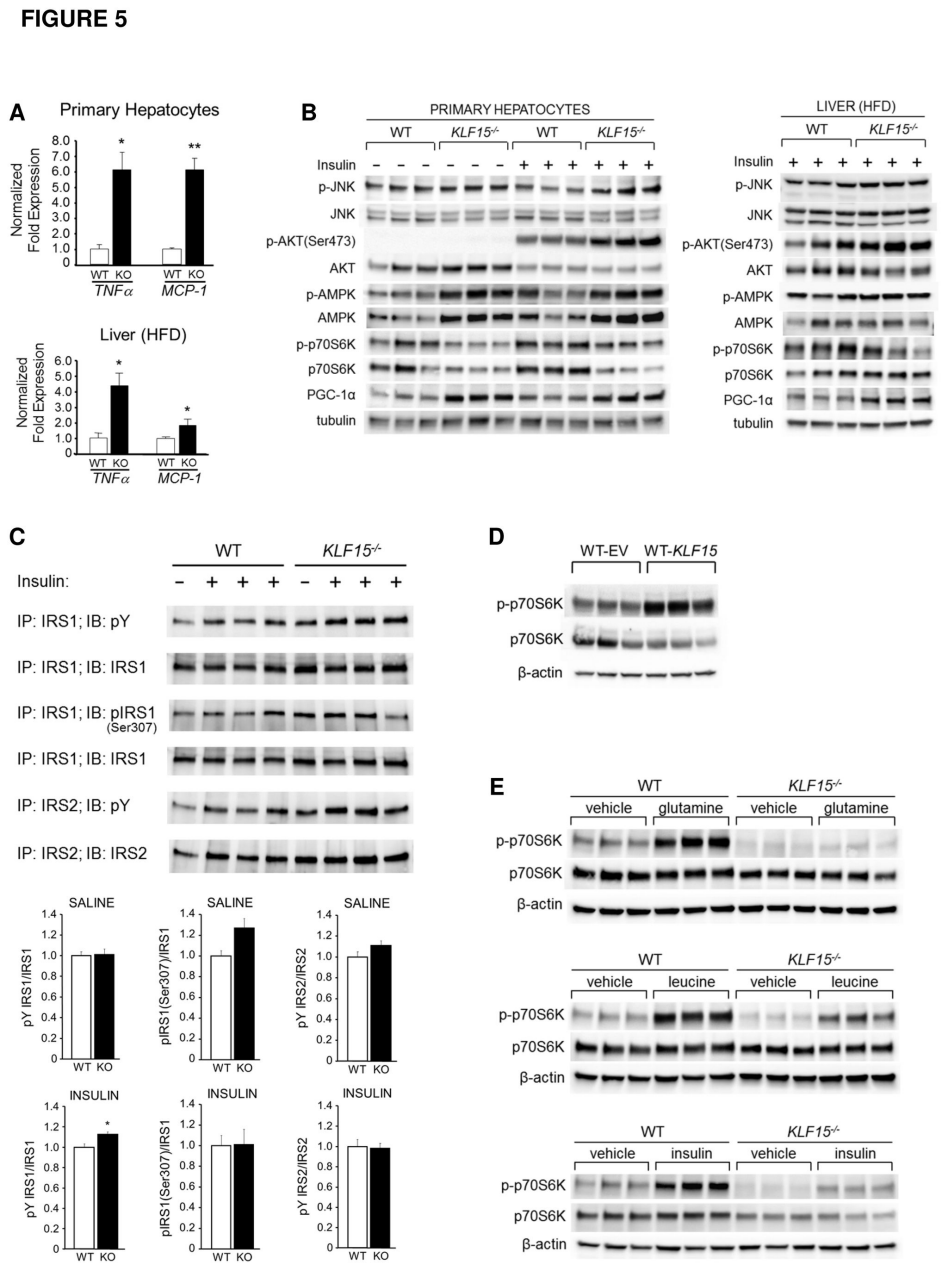
Potential mechanisms underlying the uncoupling of ER stress and insulin resistance in KLF15-deficient liver. **A**. **Inflammatory marker expression**. Total RNA was extracted from hepatocytes isolated from chow-fed 4-month-old male WT and *KLF15* ^*-/-*^ mice (top) or from liver tissues isolated from WT and *KLF15* ^*-/-*^ male mice that received HFD (60% kcal from fat) for 8 weeks starting at age 2 months, were fasted for 5h, i.p. injected with 10mU/g insulin and sacrificed 10 minutes post-injection (n=3; bottom). First strand cDNA was subjected to QPCR using primers against *TNFα* and *MCP-1*. **B**. **Activation of hepatic JNK, AKT and markers of energy availability**. Western analysis of protein lysates prepared from (left) hepatocytes that were isolated from chow-fed 3.5-month-old male WT and *KLF15* ^*-/-*^ mice and harvested 10 minutes after addition of saline or 100nM insulin, or from (right) liver tissues described in (A). **C**. **Proximal insulin signaling pathway activity**. Western analysis of IRS-1 and IRS-2 immunoprecipitated from protein lysates of liver tissues from WT and *KLF15* ^*-/-*^ male mice that received HFD (60% kcal from fat) for 8 weeks starting at age 2 months, were fasted for 5h, i.p. injected with saline or 10mU/g insulin and sacrificed 10 minutes post-injection (n=3). **D**. **Effect of KLF15 overexpression on mTORC1 activity**. Hepatocytes were isolated from standard chow-fed 2.5-month-old female WT mice and infected with EV or *KLF15*-containing adenovirus. Protein lysates were subjected to immunoblotting with the antibodies shown. **E**. **Effect of KLF15 deficiency on insulin and amino acid-mediated activation of mTORC1**. Hepatocytes were isolated from 3.5 month-old male WT and *KLF15* ^*-/-*^ mice. Following an overnight incubation in serum-free Williams *E medium* (without L-glutamine), cells were harvested after a 45-minute treatment with PBS or (top) 10mM L-glutamine, (middle) 10mM L-leucine or after a 10-minute treatment with PBS or 100nM insulin (bottom). Protein lysates were subjected to immunoblotting with the antibodies shown. Figure 5 primary hepatocyte experiments were performed twice in triplicate; each lane (in B, D and E) indicates a technical replicate. For Figure 5 liver blots, each lane represents one mouse. Statistical analysis was performed using Student’s t-test for unpaired samples. Values = mean ± SEM. *p<0.05; **p<0.01.

To further explore potential mechanisms underlying the protection against ER stress-induced insulin resistance in *KLF15* ^*-/-*^ liver, we determined whether the absence of KLF15 might inhibit activation of proinflammatory cytokines and/or JNK. As shown in Figure 5A, mRNA levels of the inflammatory markers tumor necrosis factor alpha (*TNFα*) and monocyte chemoattractant protein-1 (MCP-1) are not decreased, but increased in *KLF15* ^*-/-*^ versus WT primary hepatocytes isolated from chow-fed mice (top) and in liver tissue isolated from HFD-fed *KLF15* ^*-/-*^ versus WT mice (bottom), suggesting that inflammation and insulin resistance are uncoupled in *KLF15* ^*-/-*^ liver. We also observe an increase in phosphorylation of both JNK and AKT protein in *KLF15* ^*-/-*^ versus WT hepatocytes (Figure 5B, left) and in *KLF15* ^*-/-*^ versus WT liver from HFD-fed mice (Figure 5B, right; AKT result originally shown in Figure 2A). These results are consistent with the uncoupling of JNK activation from hepatic insulin resistance in *KLF15* ^*-/-*^ mice. We conclude that the absence of KLF15 causes increased hepatic JNK activity and inflammation independent of diet-induced metabolic effects and that KLF15 may be required for both JNK and inflammation-mediated insulin resistance in liver.

### Role of energy availability in the uncoupling of ER stress and insulin resistance in KLF15^-/-^ liver

*KLF15* ^*-/-*^ mice exhibit indicators of elevated FAO, suggesting an upregulation of catabolism for the purpose of energy production. This led us to explore the possibility that the absence of KLF15 creates a low cellular energy state. In eukaryotic cells, 5’-AMP-activated protein kinase (AMPK) is a major energy sensing molecule that is activated (via phosphorylation at threonine 172) in response to a reduction in the ratio of ATP to AMP (reviewed in 29). Consistent with a nutrient deprivation phenotype, AMPK phosphorylation is significantly increased in hepatocytes isolated from chow-fed *KLF15* ^*-/-*^ versus WT mice (Figure 5B). An important energy-sparing function of activated AMPK is to reduce protein translation by inhibiting mTORC1 [30]. As shown in Figure 5B, decreased phosphorylation of 70 kD ribosomal protein S6 kinase (p70S6K), a well-characterized target of mTORC1, provides evidence for reduction of mTORC1 activity in *KLF15* ^*-/-*^ hepatocytes. In addition to inhibiting energy-consuming pathways, phosphorylated AMPK positively regulates the expression and activity of factors involved in energy production; e.g., PGC-1α, a major regulator of mitochondrial FAO [31], [32]. Consistent with increased hepatic expression of *PGC-1α* mRNA (Figure 1D), we also see an elevation of PGC-1α protein expression in *KLF15* ^*-/-*^ versus WT hepatocytes (Figure 5B). A similar pattern of AMPK, mTORC1 and PGC-1α activity is also found in liver tissue isolated from HFD-fed *KLF15* ^*-/-*^ versus WT mice (Figure 5B, right panel). Thus, under both chow and HFD conditions, *KLF15* ^*-/-*^ liver exhibits markers of decreased energy availability. 

Interestingly, we find that PGC-1α may play a novel role in the amelioration of ER stress in the liver. WT primary hepatocytes were infected with adenovirus containing a *lacZ* control vector or a cDNA construct encoding the full-length open reading frame of *PGC-1α*. As shown in Figure S3, Western analysis revealed that overexpression of PGC-1α suppressed Tm-induced expression of total and phosphorylated PERK and IRE1α, as well as GRP78 and ATF6 (expression of the PGC-1α target CPT 1a is included as a positive control). PGC-1α also reduces ATF6 and total/phospho-PERK expression at baseline, prior to Tm induction of acute ER stress (vehicle treated). These results identify PGC-1α as a novel regulator of ER stress signaling in the liver and suggest that in *KLF15* ^*-/-*^ liver, PGC-1α induction may occur as a compensatory response to the absence of KLF15. 

### Hepatic insulin signaling pathway activity in WT versus KLF15^-/-^ mice under high-fat feeding conditions

The activation of JNK is known to inhibit insulin signaling pathway activity upstream of AKT, via serine phosphorylation of IRS-1 (insulin receptor substrate-1). Thus it is possible that increased AKT activity in *KLF15* ^*-/-*^ versus WT liver might result from an inability of JNK activation to suppress IRS-1 signaling. To investigate this, liver tissue was isolated from HFD-fed WT and *KLF15* ^*-/-*^ mice and assessed for tyrosine (activating) phosphorylation of both IRS-1 and IRS-2 and for phosphorylation of IRS-1 at serine 307, as JNK-mediated phosphorylation of this residue is associated with inhibition of insulin signaling. As shown in Figure 5C, tyrosine phosphorylation of IRS-1 and IRS-2 is markedly increased in *KLF15* ^*-/-*^ versus WT liver at baseline (saline) and after insulin-stimulation, despite increased IRS-1 serine 307 phosphorylation. Importantly, levels of IRS-1 and IRS-2 total protein are also increased in *KLF15* ^*-/-*^ versus WT liver. These data indicate that activation of IRS-1 and IRS-2 is increased in *KLF15* ^*-/-*^ versus WT liver primarily as a result of increased total protein levels in *KLF15* ^*-/-*^ cells, rather than through increased tyrosine phosphorylation per molecule of IRS-1 or IRS-2. However, Figure 5C quantitation graphs also indicate a modest, but statistically significant increase in tyrosine phosphorylation per molecule of IRS-1 in insulin-stimulated *KLF15* ^*-/-*^ versus WT liver, with no significant difference in tyrosine phosphorylation per molecule of IRS-2 or serine 307 phosphorylation per molecule of IRS-1 between groups. Our results suggest that in *KLF15* ^*-/-*^ liver, JNK activity and ER stress are uncoupled from IRS-1 inhibition. It is possible that the amount of serine 307 phosphorylation per molecule of IRS-1 in *KLF15* ^*-/-*^ liver is not sufficient to reduce activation of IRS-1 to a level below that of WT liver. Alternatively, phosphorylation of serine 307 may be necessary, but insufficient to inhibit IRS-1 activation. Furthermore, a recent study indicates that phosphorylation of IRS-1 at serine 307 can promote insulin sensitivity, as mice containing an IRS-1 serine 307 to alanine mutation were more insulin resistant than control mice after high-fat feeding [33].

### Potential mechanisms underlying KLF15 regulation of mTORC1 activity

mTORC1 has been shown to promote insulin resistance through mechanisms that include negative feedback inhibition of AKT [34] and activation of the JNK/IRE pathway, leading to increased ER stress [35]. Consistent with our finding that KLF15 deficiency decreases mTORC1 activity, adenoviral overexpression of KLF15 in WT primary hepatocytes results in increased phosphorylation of p70S6K, indicating enhanced mTORC1 activation (Figure 5D). Insulin and amino acids, particularly glutamine and leucine, are critical activators of mTORC1 [36], [37]. Because KLF15 is known to regulate amino acid catabolism [24], we reasoned that KLF15 might be involved in amino acid regulation of mTORC1 activity. As shown in Figure 5E, phosphorylation of p70S6K in response to saturating concentrations (10mM) of either L-glutamine or L-leucine is decreased in *KLF15* ^*-/-*^ versus WT hepatocytes. This suggests that protection against amino acid-mediated activation of mTORC1 may be one mechanism by which insulin sensitivity is maintained in *KLF15* ^*-/-*^ liver. This mechanism may take on increased significance during high-fat feeding, when catabolism of branched-chain amino acids, including leucine, is known to promote insulin resistance in an mTORC1-dependent manner [38]. Activation of mTORC1 by insulin is also inhibited in *KLF15* ^*-/-*^ versus WT hepatocytes (Figure 5E, bottom panel; results shown are for serum-starved cells. Similar results (Figure 5B, left panel) for phosphorylation of p70S6K in response to insulin were achieved in WT and *KLF15* ^*-/-*^ hepatocytes grown in complete medium). Further work will determine whether this inhibition results from a block in amino acid-mediated activation of mTORC1 in *KLF15* ^*-/-*^ liver, as prior studies indicate that amino acids are required for insulin stimulation of mTORC1 [39].

### KLF15 regulation of autophagy

**Figure 6:**
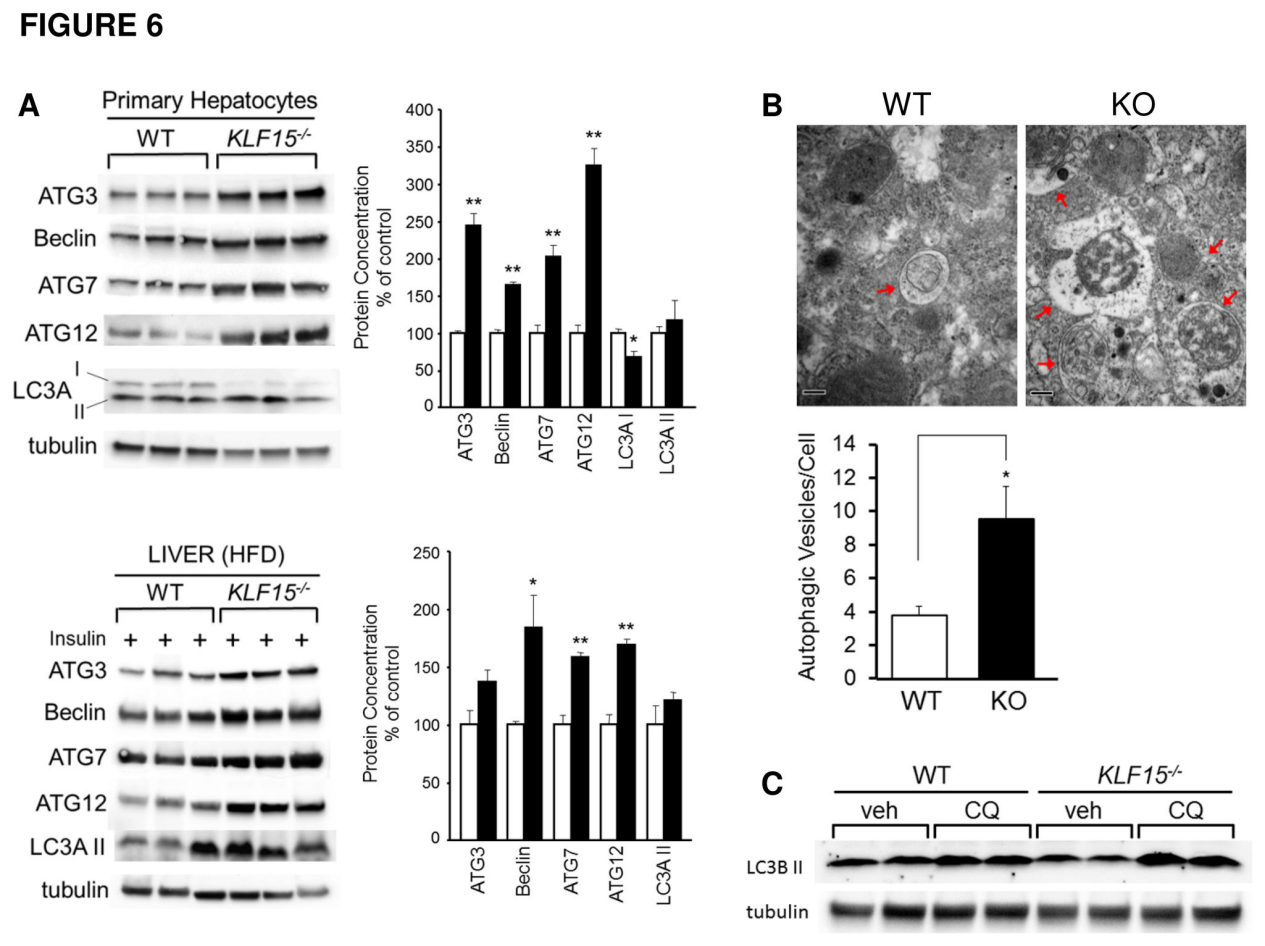
KLF15 regulation of autophagy. **A**. **Autophagy marker expression in WT and *KLF15*^*-/-*^ primary hepatocytes and liver tissue**. (Top) Hepatocytes were isolated from standard chow-fed 4-month-old male WT and *KLF15* ^*-/-*^ mice. Protein lysates were subjected to immunoblotting with the antibodies shown. (Bottom) 2-month-old WT and *KLF15* ^*-/-*^ male mice were placed on a HFD (60% kcal from fat) for 8 weeks. Mice were fasted for 5h, then i.p. injected with 10mU/g insulin and sacrificed 10 minutes post-injection. Lysates from flash frozen livers were subjected to immunoblotting with the antibodies shown (n=3). Quantitation graphs are shown next to immunoblots (white bar = WT, black bar = KLF15^-/-^). **B**. **Ultrastructural examination of autophagic vesicles in liver tissue from HFD-fed WT and *KLF15*^*-/-*^ mice**. WT and *KLF15* ^*-/-*^ female mice (n=3) received HFD (60% kcal from fat) for 4 weeks starting at age 3 months. Mice were fasted for 21h before sacrifice and liver tissue was removed and prepared for transmission electron microscopy (TEM) analysis as described in Materials and Methods. (Top) Electron micrographs (original magnification = 43,000x) of liver sections showing clustering of autophagic vesicles (indicated by red arrows) in *KLF15* ^*-/-*^ versus WT hepatocytes. Scale bar = 200nm. (Bottom) Quantitation of autophagic vesicles in WT and *KLF15* ^*-/-*^ liver. TEM was used to visualize autophagic vesicles in intact hepatocytes. Values = number of autophagic vesicles per cell calculated from 15 hepatocytes per group. **C**. **Chloroquine treatment of primary hepatocytes**. Hepatocytes were isolated from 3.5-month old male WT and *KLF15* ^*-/-*^ mice and treated with vehicle or 50µM chloroquine (CQ) for 2h prior to harvest. Protein lysates were subjected to immunoblotting with an antibody against LC3B. Figure 6 primary hepatocyte experiments were performed at least twice in triplicate; each lane indicates a technical replicate. For Figure 6A liver blot, each lane represents one mouse. Statistical analysis was performed using Student’s t-test for unpaired samples. Values = mean ± SEM. *p<0.05; **p<0.01.

Activation of autophagy is often an indicator of nutrient starvation [40]. In response to starvation, sugars, fatty acids and amino acids are made available through autophagic degradation for the production of ATP. Over the past decade, numerous autophagy-related genes (*ATG*-genes) have been identified that regulate the mammalian autophagic machinery (reviewed in 41). Here we find that *KLF15* ^*-/-*^ versus WT hepatocytes isolated from chow-fed mice show increased expression of the *ATG*-genes, *ATG3*, *Beclin* (*ATG6*), *ATG7*, *ATG12* and microtubule-associated protein 1 light chain 3 (LC3), which are involved in the formation of the double-membrane vesicle, or autophagosome that surrounds cytoplasmic contents prior to fusion with the lysosome (Figure 6A, top). It should be mentioned that there are at least 3 mammalian LC3 isoforms (LC3A, LC3B and LC3C), and LC3 proteins are known to be post-translationally modified, resulting first in LC3-I, which is processed into a faster migrating form, LC3-II, that associates with the autophagosome membrane [42]. Thus, decreasing levels of LC3-I and increasing levels of LC3-II are indicative of autophagosome formation. As shown in Figure 6A (top), the ratio of LC3A-II to LC3A-I is higher in *KLF15* ^*-/-*^ versus WT hepatocytes. We also find increased expression of autophagic markers in *KLF15* ^*-/-*^ versus WT liver isolated from HFD-fed mice (Figure 6A, bottom; LC3A-I was undetectable in this analysis, although LC3A-II expression trended upward in *KLF15* ^*-/-*^ versus WT liver). Using transmission electron microscopy, we also observe an increase in the quantity of autophagic vesicles in liver tissue isolated from *KLF15* ^*-/-*^ versus WT mice after high-fat feeding (Figure 6B, bottom). Furthermore, autophagic vesicles were frequently found in clusters in *KLF15* ^*-/-*^ hepatocytes, but appeared scattered in WT hepatocytes (Figure 6B, top). Korolchuk et al. have recently shown that nutrient deprivation causes lysosomes to form clusters in the perinuclear region of the cell, thereby inactivating mTORC1 and promoting autophagosome-lysosome fusion [43]. Clustering of autophagic vesicles in *KLF15* ^*-/-*^ hepatocytes may therefore indicate increased autophagic flux and also suggest a potential mechanism for low energy state-mediated reduction of mTORC1 activity in the absence of KLF15.

**Figure 7:**
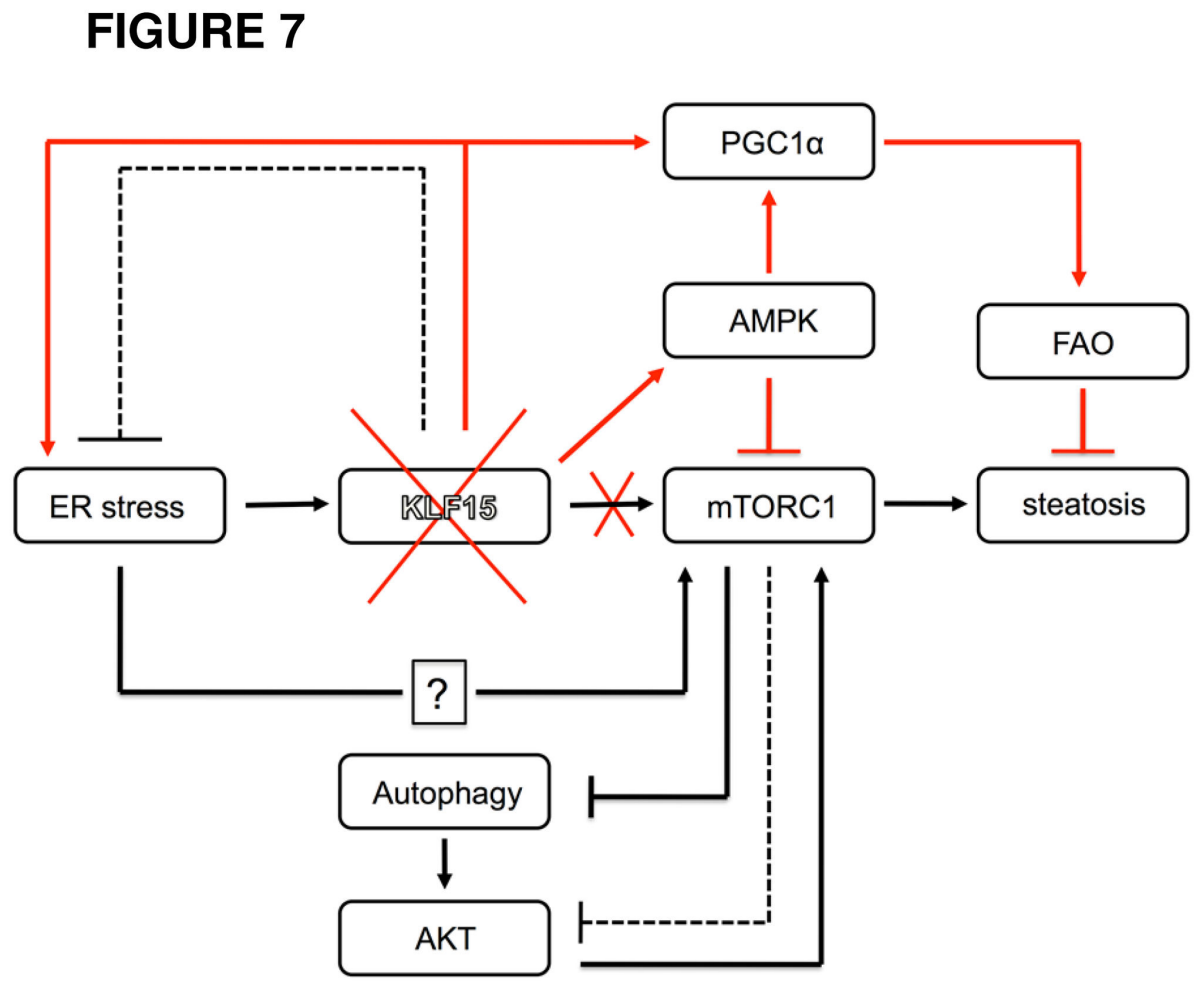
Proposed model for KLF15 regulation of ER stress-mediated insulin resistance. The diagram places KLF15 into the network of ER stress, mTORC1 and autophagy signaling pathways that regulate the onset of insulin resistance (red lines indicate pathways that are activated with KLF15 deficiency; dotted lines represent negative feedback inhibition). Our data indicate that acute ER stress induces KLF15 and that KLF15 activates mTORC1. Previous studies have shown that ER stress can induce mTORC1, although the mechanisms by which this occurs have not been fully delineated. Activation of mTORC1 leads to inhibition of autophagy as well as feedback inhibition of AKT. Because KLF15 overexpression reduces UPR signaling, it is hypothesized that 1) induction of KLF15 in response to ER stress may serve to reduce ER stress levels through negative feedback inhibition and 2) as ER stress is resolved, KLF15 would no longer be induced and mTORC1 activity would decrease, allowing for increased signaling through AKT. The absence of KLF15 results in ER stress and inflammation, but also activation of AMPK and reduced mTORC1 activity, preventing mTORC1 pathway-mediated inhibition of AKT. Loss of KLF15 also leads to elevated PGC-1α expression, potentially resulting in increased fatty acid oxidation (FAO), which may indirectly promote insulin sensitivity by reducing hepatic lipid content.

While increased autophagic vesicle numbers and expression of ATG-genes generally indicate enhanced autophagic activity, the possibility still exists that our data reflect a block in lysosomal degradation, rather than accelerated formation of autophagosomes. To assess this, we treated WT and *KLF15* ^*-/-*^ hepatocytes with chloroquine (CQ), an agent that blocks the activity of lysosomal hydrolases and reduces autophagosome-lysosome fusion. As shown in Figure 6C, the rise in LC3B-II content in CQ-treated versus untreated *KLF15* ^*-/-*^ hepatocytes is higher than that in WT hepatocytes. This suggests an increase in autophagic flux and also that the slight decrease in LC3B-II content in untreated *KLF15* ^*-/-*^ versus WT hepatocytes may indicate a more rapid turnover of autophagosomes. These results provide additional evidence that *KLF15* ^*-/-*^ mice maintain a low energy state compared to WT controls, even under high-nutritional conditions. In addition, the maintenance of increased autophagic flux may be a mechanism by which *KLF15* ^*-/-*^ mice are protected against high-fat diet-induced insulin resistance. Finally, Figure 7 shows a proposed model of the mechanisms involved in KLF15 regulation of ER stress-mediated insulin resistance based upon our current findings.

## Discussion

A key finding in this study is that KLF15 deficiency results in the uncoupling of hepatic ER stress and insulin resistance. Increased UPR activity in *KLF15* ^*-/-*^ versus WT liver and primary hepatocytes as well as ultrastructural examination of the ER provide evidence that the absence of KLF15 results in chronic ER stress. However, *KLF15* ^*-/-*^ mice are protected against ER stress-induced hepatic insulin resistance and steatosis compared to WT controls. Our data highlight the importance of mTORC1 signaling in ER stress-mediated insulin resistance. ER stress plays an important role as an upstream activator of mTORC1 [35], [44]. In addition, a number of studies show that increased mTORC1 activity promotes ER stress and insulin resistance and that inhibition of mTORC1 ameliorates both of these conditions ([44], [45] [46],). In *KLF15* ^*-/-*^ versus WT liver, mTORC1 activity and insulin resistance are decreased, yet this is not associated with a reduction in ER stress. This raises the possibility that hepatic insulin resistance does not occur in response to ER stress per se, but rather as a result of cooperation between the UPR and mTORC1 signaling pathways. Our data suggest that the ability of *KLF15* ^*-/-*^ mice to maintain a low energy state, particularly under high-fat feeding conditions, prevents the upregulation of mTORC1 activity and inhibition of autophagy that, in concert with ER stress signaling, lead to hepatic insulin resistance.

ER stress has been implicated in the development of hepatic steatosis [47], and reduction of ER stress in rodents has been shown to decrease hepatic lipid content (reviewed in 48). In the current study, we have found that *KLF15* ^*-/-*^ mice are protected against HFD and ER stress-mediated steatosis and it is likely that increased AMPK activity in *KLF15* ^*-/-*^ liver contributes to this phenotype through inhibition of mTORC1 activity and induction of PGC-1α expression. mTORC1 plays an important role in promoting hepatic steatosis through its control of SREBP, a master regulator of lipogenesis, and inhibition of mTORC1 protects against HFD-induced hepatic lipid accumulation [49], [50]. Increased PGC1α expression in *KLF15* ^*-/-*^ liver may also play a causal role in protection against steatosis through enhancement of mitochondrial FAO. Indeed, studies in ATF6-knockout mice show that increased hepatic lipid accumulation caused by ER stress can result from blockage of β-oxidation of fatty acids [51]. In addition to observing increased hepatic expression of PGC-1α in *KLF15* ^*-/-*^ mice, we also find that PGC-1α suppresses ER stress induction of UPR activity in WT primary hepatocytes. In contrast, PGC-1α recently has been shown to cooperate with ATF6 to induce UPR genes in skeletal muscle in response to exercise [52]. This tissue-specific regulation of the UPR by PGC-1α is not unexpected, given the different roles of UPR signaling in liver versus skeletal muscle. 

mTORC1 activity depends largely upon signals emanating from energy levels, insulin and amino acids. Insulin stimulation of mTORC1 activity is known to occur through the PI3K/AKT pathway. Phosphorylation of the GTPase-activating protein, tuberous sclerosis complex 2 (TSC2), by AKT prevents TSC2 from inhibiting the small GTPase Rheb, an activator of mTORC1 (reviewed in 53). Insulin is also thought to activate mTORC1 via AKT-mediated phosphorylation of an inhibitory binding partner of mTOR termed proline-rich Akt/PKB substrate 40 kDa, or PRAS40 [54]. Amino acids appear to use a distinct pathway for the activation of mTORC1 that is mediated by the Rag family of small GTPases [55]. Recent evidence shows that amino acids stimulate Rag GTPase-mediated translocation of mTORC1 to lysosomes, where mTORC1 is then activated by Rheb [56]. These data may explain previous studies indicating that amino acids are required for activation of mTORC1 by insulin [39]. Here we find that activation of mTORC1 is markedly inhibited in *KLF15* ^*-/-*^ versus WT hepatocytes in response to insulin and to the critical regulatory amino acids, leucine and glutamine. Although the underlying mechanism is yet to be defined, the previous identification of KLF15 as a regulator of amino acid catabolism [24] suggests the possibility that KLF15 is a specific effector of amino acid-mediated mTORC1 activation; inhibition of insulin-mediated mTORC1 activation in *KLF15* ^*-/-*^ hepatocytes would then occur as a secondary effect. In addition, consistent with impaired amino acid utilization, *KLF15* ^*-/-*^ mice are unable to maintain nitrogen homeostasis, exhibiting hyperammonemia and defective ureagenesis [26]. Thus, given the sensitivity of mTORC1 to the availability of nitrogen sources [57], the inability of *KLF15* ^*-/-*^ mice to appropriately metabolize nitrogen may contribute to the inhibition of mTORC1 in response to amino acids.

While the mechanism behind the maintenance of a low energy state in *KLF15* ^*-/-*^ mice has not been resolved, we reasoned that it results from either an inability to generate sufficient ATP or from increased energy expenditure (consumption), or a combination of both. Figure 1B shows that high-fat feeding reduces energy expenditure in both WT and *KLF15* ^*-/-*^ mice, but to a greater extent in WT mice. We attribute this to the fact that WT mice gain significantly more fat mass than *KLF15* ^*-/-*^ mice in response to a HFD. Importantly, we know that under chow conditions, while energy expenditure is not significantly different between groups, *KLF15* ^*-/-*^ mice already show indications of a nutrient starvation phenotype, as evidenced by increased PGC-1α expression and phosphorylation of AMPK, decreased mTORC1 activity and activation of autophagy. This suggests that the inability of *KLF15* ^*-/-*^ mice to generate ATP, rather than increased energy expenditure, is responsible for their low energy state. Our previous work shows that *KLF15* ^*-/-*^ mice utilize amino acids less efficiently than WT mice, resulting in limited substrate for gluconeogenesis [24]. Thus, defective amino acid catabolism is likely to play an important role in keeping the AMP/ATP ratio high in *KLF15* ^*-/-*^ liver, causing activation of AMPK. 

Defective amino acid catabolism may be promoting ER stress in *KLF15* ^*-/-*^ mice through several mechanisms. First, circulating levels of several amino acids are increased in *KLF15* ^*-/-*^ mice [24]. This amino acid surplus could potentially contribute to a chronic increase in protein translation that, over time, may result in accumulation of misfolded proteins and ER dysfunction. Also, inefficient use of amino acids for ATP production may cause *KLF15* ^*-/-*^ mice to increase their use of glucose as an alternative energy source. Indeed, *KLF15* ^*-/-*^ mice exhibit decreased plasma glucose levels in the fed state and hypoglycemia after an overnight fast [24]. This could contribute to ER stress by causing glycosylation defects that lead to protein misfolding. 

Finally, the process of autophagy has been the focus of much attention recently for its role in the regulation of a variety of physiological and pathological states, including cancer, neurodegenerative disorders, aging and heart disease. It will be important to further investigate the mechanisms by which KLF15 controls autophagy in the liver and in other tissues. In conclusion, this work identifies KLF15 as a critical regulator of ER stress, mTORC1 and autophagy signaling in the liver and underscores the importance of this factor in the maintenance of energy balance and glucose homeostasis.

## Materials and Methods

### Animals

Mouse studies were approved by the Institutional Animal Care and Use Committee at the University of Massachusetts Medical School (Permit Number: A-2065), and all efforts were made to minimize discomfort. *KLF15* ^*-/-*^ mice backcrossed onto a C57BL/6 background were generated and genotyped as previously described [58]. Mice were housed under pathogen-free conditions and maintained on a 12-hour light-dark cycle with free access to standard chow (ProLab Isopro RMH 3000, LabDiet) and water. High-fat fed mice received a diet containing 60% kcal from fat (Research Diets #D12492).

### Reagents

A cDNA containing the full-length open reading frame of *KLF15* (pcDNA3.1-*KLF15* FLAG) was subcloned into an expression vector as previously described [58]. Empty vector (EV) control and pcDNA3.1-*KLF15* FLAG adenoviral vectors were prepared by Applied Viromics, LLC (Fremont, CA). The *PGC-1α* and control *lacZ* adenoviral vectors were kindly provided by the laboratory of Dr. Bruce Spiegelman. The shControl and sh*KLF15* adenoviral vectors were a gift from the laboratory of Dr. Mukesh Jain.

### Hyperinsulinemic-euglycemic clamps

A 2 hour hyperinsulinemic-euglycemic clamp was conducted with a primed (150 mU/kg body weight) infusion of human insulin followed by a continuous infusion of insulin at a rate of 2.5 mU/kg/min to raise and maintain plasma insulin within a physiological range (~300 pM). Blood samples (20 μl) were collected at 10-20 min intervals for the immediate measurement of plasma glucose concentration, and 20% glucose was infused at variable rates to maintain glucose at basal concentrations (~6mM). Insulin-stimulated whole body glucose metabolism was assessed with a continuous infusion of [3-^3^H]glucose (0.1 mCi/min) throughout the clamp. Clamp hepatic glucose production was calculated as the difference between whole body glucose turnover and glucose infusion rate during the insulin clamp.

### Body composition analysis and metabolic cages

Measurement of food intake, VO_2_ consumption, VCO_2_ production and physical activity over a 3-day period was performed using metabolic cages (TSE Systems, Bad Homburg, Germany). Whole-body fat mass was noninvasively measured in conscious mice using 1H-MRS (Echo Medical Systems, Houston, TX).

### Immunoblotting

Flash-frozen tissues were ground in liquid nitrogen and sonicated in lysis buffer containing 50mM HEPES pH 7.4, 100mM sodium pyrophosphate, 10mM EDTA, 100mM sodium fluoride, 10mM sodium orthovanadate, 2mM PMSF and 1% Triton X-100. Protease inhibitor cocktail (SIGMA P2714) and phosphatase inhibitor cocktails 2 and 3 (SIGMA P5726, P0044) were added as recommended by the manufacturer. Hepatocytes were harvested in NP40 Cell Lysis Buffer (Invitrogen FNN0021) supplemented with 1mM PMSF and protease inhibitor cocktail (SIGMA P2714), phosphatase inhibitor cocktails 2 and 3 (SIGMA P5726, P0044) as recommended by the manufacturer. Lysates were rotated at 4°C for 1 hour, centrifuged at 18,000 x g for 2 minutes and supernatant was assayed for protein content using a kit from Pierce (Thermo Scientific #23227). Proteins were resolved by SDS-PAGE and transferred to nitrocellulose membranes (Hybond ECL, RPN2020D). Immunoblotting was performed using antibodies against AKT, p-AKT(Ser 473), p-AKT(Thr308), PERK, p-PERK (Thr980), eIF2α, p-eIF2α (Ser51), IRE1α and GRP78 (BiP), p70 S6 Kinase, 4E-BP1, p-4E-BP1(Ser65), AMPK, p-AMPK (Thr172), ATG3, Beclin, ATG7, ATG12, LC3A, LC3B, β-actin and α-Tubulin (Cell Signaling Technology), ATF6, GAPDH and ALT1(GPT) (Santa Cruz Biotechnology), p-JNK (Thr183/Tyr185, Thr221/Tyr223), p-p70 S6 Kinase (Thr389) and PGC-1 (Millipore), CPT 1a and KLF15 (ProteinTech Group, Inc.), p-IRE1α (Novus Biologicals) and SAPK/JNK (Calbiochem). Anti-rabbit or mouse IgG, HRP-linked secondary antibodies were purchased from Cell Signaling Technology. Immunoreactive band intensity was quantified on the FluorChem HD2 System using AlphaEase densitometry software (Alpha Innotech, San Leandro, CA). 

### Immunoprecipitation of IRS-1/IRS-2

Liver lysates were diluted to 2µg/ul protein in 500µl of tissue lysis buffer. Lysates were precleared by adding 25µl of protein A/G agarose (Pierce #20421) and 3µl normal rabbit IgG (Santa Cruz Biotechnology) followed by 1h rotation at 4C. Samples were centrifuged at 10K RPM for 1 min at 4C to pellet agarose beads, and supernatant was transferred to new tubes. 2µg of IRS-1 or IRS-2 antibody (gifts from laboratory of Leslie Shaw) and 25µl of protein A/G agarose were added to supernatant, followed by O/N rotation at 4C. Beads were pelleted and washed 3x in 400µl tissue lysis buffer. After final wash, lysis buffer was removed and 25µl of 2x sample buffer was added to bead pellet. Samples were boiled for 5 min and subjected to gel electrophoresis. Proteins resolved by SDS-PAGE were transferred to a nitrocellulose membrane and immunoblotting was performed using antibodies against total IRS-1, phosphorylated tyrosine (PY99) (Santa Cruz Biotechnology) and phosphorylated IRS-1(Ser307), total IRS-2 (Cell Signaling). 

### Real-Time Quantitative PCR (qPCR)

Mouse gene-specific primers were selected from the PrimerBank database [59] or were user-designed (sequences listed in Table S1). Total RNA was isolated from cells or tissues using TRIzol Reagent (Life Technologies) according to manufacturer’s instructions and reverse transcribed using an iScript Advanced cDNA Synthesis Kit (BioRad). An iQ SYBR Green Supermix Kit (BioRad) was used for cDNA amplification with a CFX96 Real-Time PCR Detection System (BioRad). Expression values were normalized against hypoxanthine guanine phosphoribosyl transferase (HPRT) and beta-2 microglobulin (B2m), (Figure 1D); HPRT and TATA-box binding protein (TBP), (Figures 2D, 5A) or HPRT (Figure 3C). 

### Electron microscopy

Transmission electron microscopy was performed using tissue washed with 0.1 M sodium cacodylate buffer (pH 7.2), and fixed with 2.5% gluteraldehyde in the same buffer, for 30 min at room temperature and in the same fresh fixative overnight at 4°C. The tissue was then rinsed for 10 min in 0.1 M sodium cacodylate buffer (pH 7.2) 3 times and post-fixed (1h) in 1% (wt/vol) osmium tetroxide in distilled water. The fixed tissue was rinsed in double-distilled water and dehydrated through a graded ethanol series before two changes of 100% ethanol. Samples were then infiltrated with 2 changes of 100% propylene oxide and then with a 50%/50% propylene oxide / SPI-Pon 812 resin mixture. The following day, 3 changes of fresh 100% SPI-Pon 812 resin were done before the samples were polymerized at 68°C in flat embedding molds. The epoxy blocks were cut and mounted on blank epoxy stubs with a drop of Super Glue, and ultrathin sections were cut on a Reichart-Jung ultramicrotome using a diamond knife. The sections were collected and mounted on copper support grids, contrasted with lead citrate and uranyl acetate, and examined using an FEI Tecnai 12 BT with 80Kv accelerating voltage. Images were captured using a Gatan TEM CCD camera. ER lumen diameters were measured using Gatan DigitalMicrograph Software.

### Metabolic measurements

Blood glucose values were measured using a One Touch Ultra glucometer and test strips (Lifescan). For GTT, mice were fasted overnight and received an intraperitoneal injection of 1g glucose/kg body weight. Tail vein blood samples were assessed for glucose concentration immediately before injection (Time 0) and at 15, 30, 60 and 120 minutes post-injection. Plasma insulin concentration was determined using an Ultrasensitive Mouse Insulin ELISA Kit (Crystal Chem, Inc.). A Triglyceride Quantification Kit (MBL International) was used to measure lipid content in frozen liver samples. 

### Histological analysis

Frozen liver tissues were sectioned and treated with hematoxylin nuclear stain and Oil Red O for detection of lipid at the Morphology Core Facility at UMass Medical School.

### Cell culture

The AML12 (alpha mouse liver 12) hepatocyte cell line was purchased from the American Type Culture Collection and grown in a 1:1 mixture of Dulbecco's modified Eagle's medium and Ham's F12 medium (Mediatech #10-090-CV) with 10% FBS, 100U/ml penicillin, 100µg/ml streptomycin, 1X Insulin-Transferrin-Selenium (ITS) supplement (Mediatech #25-800-CR) and 40 ng/ml dexamethasone. Cells were plated at 5 x 10^5^ cells per well in 6-well plates one day prior to treatment with Tm or Tg. Primary hepatocytes were isolated via liver perfusion (see Protocol S1). Cells were plated at 1 x 10^6^ cells per well in 6-well collagen-coated plates and grown in William’s Medium E (Life Technologies, #12551032) supplemented with 10% FBS, 100U/ml penicillin, 100µg/ml streptomycin, 1nM insulin, 40ng/ml dexamethasone and 5µg/ml transferrin.

### Statistical Analysis

Values are expressed as means ± SEM. Comparisons between groups were made using a two-tailed Student’s t test for unpaired samples or a repeated measures ANOVA with a Bonferroni post hoc test where appropriate. Differences were considered statistically significant at p < 0.05.

## Supporting Information

Figure S1 **Glucose tolerance test.** WT and KLF15^-/-^ (KO) male mice were placed on a high-fat diet (HFD; 60% kcal from fat) at 3-4 months of age. After 8 weeks of HFD, mice were fasted for 16h and received an intraperitoneal injection of 1g glucose/kg body weight. Tail vein blood samples were assessed for glucose concentration immediately before injection (Time 0) and at 15, 30, 60 and 120 minutes post-injection (n=6-7). Left: blood glucose concentrations during the GTT. Middle: area under the curve calculations for glucose values. Right: plasma insulin concentrations during the GTT. Plasma isolated from tail vein blood samples collected at Time 0 and at 15, 30 and 60 minutes after glucose injection was assayed for insulin concentration using an ELISA kit. Statistical comparisons were made using Student’s t test for unpaired samples (AUC) or analysis of variance for repeated measures with a Bonferroni post hoc test (GTT/Insulin during GTT). Values = mean ± SEM; **p<0.01 compared to WT control.
(TIF)

Figure S2 **Plasma ketone measurements.** Male WT and *KLF15* ^*-/-*^ (KO) mice were placed on a high-fat diet (HFD; 60% kcal from fat) at 3-4 months of age. 3-hydroxybutyrate levels were measured in whole blood from overnight-fasted and ad libitum fed mice, 9 and 10 weeks, respectively, after the start of HFD using a MediSense Precision Xtra Monitor with β-Ketone Test Strips (Abbott Laboratories). n=6. Statistical comparisons were made using Student’s t test for unpaired samples. Values = mean ± SEM; **p<0.01 compared to WT control.
(TIF)

Figure S3 **Adenoviral overexpression of PGC-1α in primary hepatocytes.** Primary hepatocytes isolated from 3.5-month-old chow-fed WT male mice were infected with adenovirus containing *PGC-1α* or *lacZ* control. Cells were harvested after 20h treatment with vehicle (DMSO) or 2µg/ml Tm. Protein lysates were subjected to immunoblotting with the antibodies shown. A quantitation graph is shown below the blot: protein expression levels were normalized against β-actin, α-tubulin or GAPDH. Statistical analysis was performed using Student’s t-test for unpaired samples. Values = mean ± SEM of triplicate values, representative of two individual experiments. *p<0.05; **p<0.01. # = p<0.07; Statistical comparisons refer to *PGC-1α* vehicle versus *lacZ* vehicle or to *PGC-1α*-Tm versus *lacZ*-Tm.
(TIF)

Protocol S1 **Description of procedure for liver perfusion and primary hepatocyte isolation in mice.**
(DOC)

Table S1 **QPCR Primer List, Related to Figures 1D, 2D, 3C and 5A.**
(DOC)
