# Supplementary figures and images for: KLF15 Is a Molecular Link between Endoplasmic Reticulum Stress and Insulin Resistance
Source: PLoS One. 2013 Oct 22;8(10):e77851. doi: 10.1371/journal.pone.0077851 (PMC3805598; doi:10.1371/journal.pone.0077851)

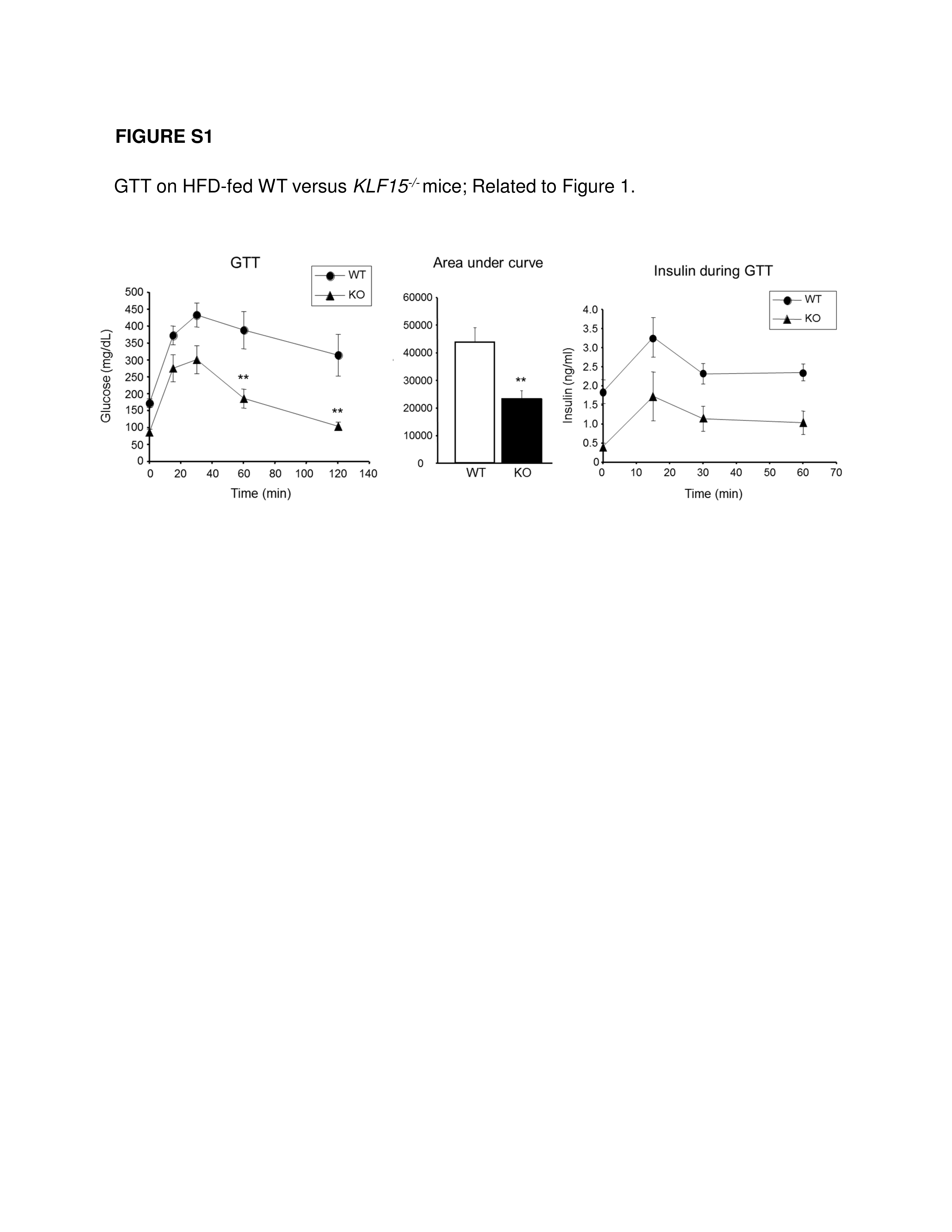

Supplement: Figure S1 — Glucose tolerance test. WT and KLF15-/- (KO) male mice were placed on a high-fat diet (HFD; 60% kcal from fat) at 3-4 months of age. After 8 weeks of HFD, mice were fasted for 16h and received an intraperitoneal injection of 1g glucose/kg body weight. Tail vein blood samples were assessed for glucose concentration immediately before injection (Time 0) and at 15, 30, 60 and 120 minutes post-injection (n=6-7). Left: blood glucose concentrations during the GTT. Middle: area under the curve calculations for glucose values. Right: plasma insulin concentrations during the GTT. Plasma isolated from tail vein blood samples collected at Time 0 and at 15, 30 and 60 minutes after glucose injection was assayed for insulin concentration using an ELISA kit. Statistical comparisons were made using Student’s t test for unpaired samples (AUC) or analysis of variance for repeated measures with a Bonferroni post hoc test (GTT/Insulin during GTT). Values = mean ± SEM; **p<0.01 compared to WT control. (TIF) [file pone.0077851.s001.tif]

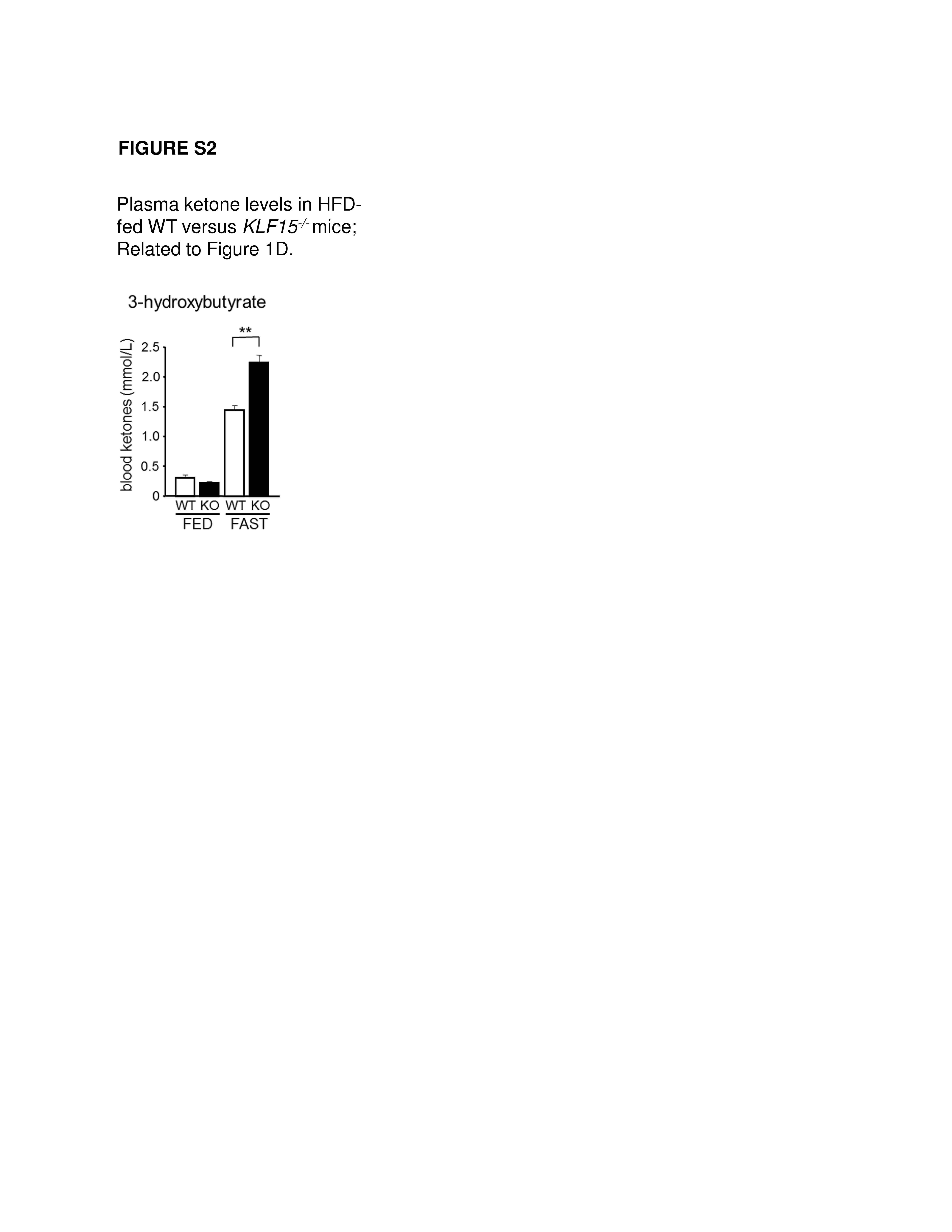

Supplement: Figure S2 — Plasma ketone measurements. Male WT and KLF15 -/- (KO) mice were placed on a high-fat diet (HFD; 60% kcal from fat) at 3-4 months of age. 3-hydroxybutyrate levels were measured in whole blood from overnight-fasted and ad libitum fed mice, 9 and 10 weeks, respectively, after the start of HFD using a MediSense Precision Xtra Monitor with β-Ketone Test Strips (Abbott Laboratories). n=6. Statistical comparisons were made using Student’s t test for unpaired samples. Values = mean ± SEM; **p<0.01 compared to WT control. (TIF) [file pone.0077851.s002.tif]

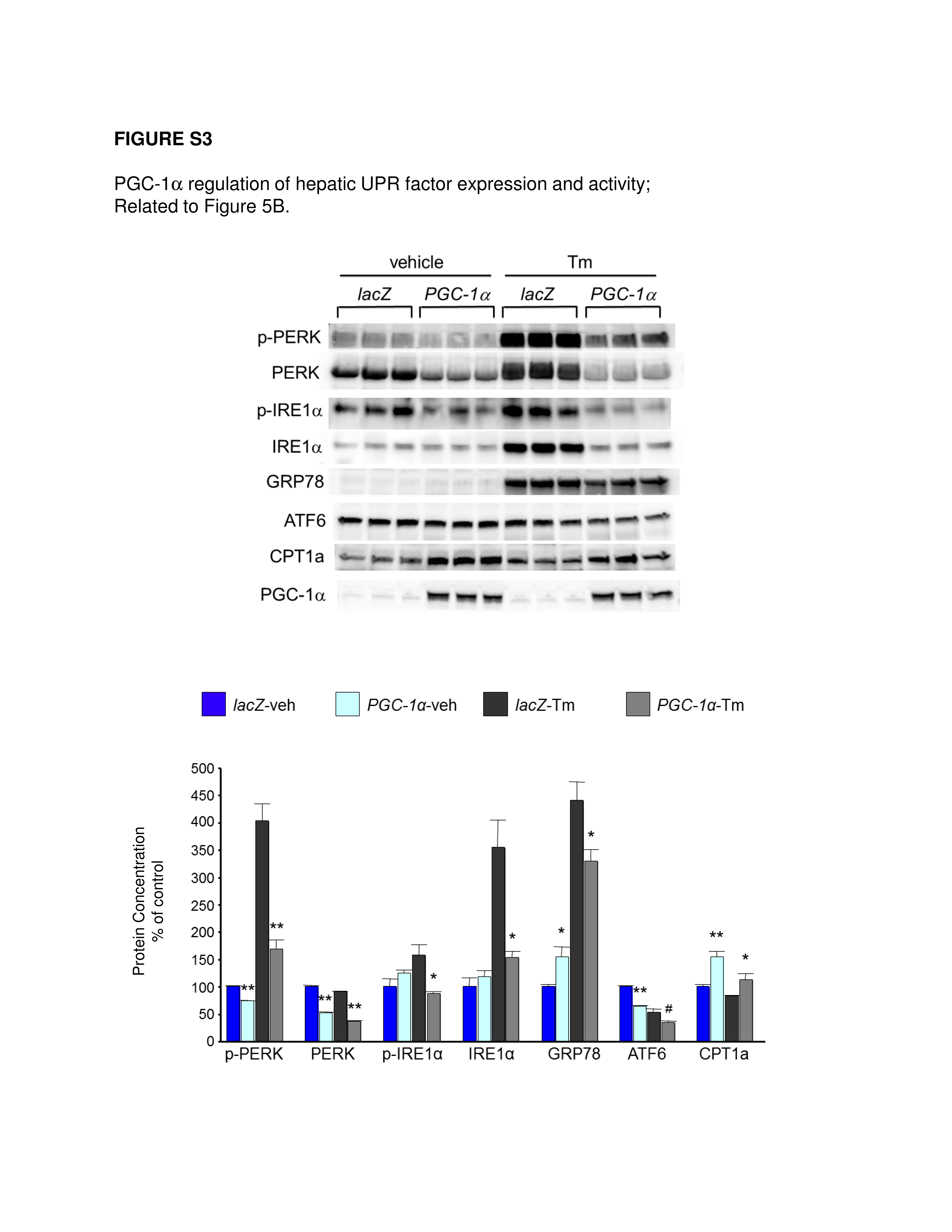

Supplement: Figure S3 — Adenoviral overexpression of PGC-1α in primary hepatocytes. Primary hepatocytes isolated from 3.5-month-old chow-fed WT male mice were infected with adenovirus containing PGC-1α or lacZ control. Cells were harvested after 20h treatment with vehicle (DMSO) or 2µg/ml Tm. Protein lysates were subjected to immunoblotting with the antibodies shown. A quantitation graph is shown below the blot: protein expression levels were normalized against β-actin, α-tubulin or GAPDH. Statistical analysis was performed using Student’s t-test for unpaired samples. Values = mean ± SEM of triplicate values, representative of two individual experiments. *p<0.05; **p<0.01. # = p<0.07; Statistical comparisons refer to PGC-1α vehicle versus lacZ vehicle or to PGC-1α-Tm versus lacZ-Tm. (TIF) [file pone.0077851.s003.tif]
