# Supplementary material for: KLF15 Is a Molecular Link between Endoplasmic Reticulum Stress and Insulin Resistance
Source: PLoS One. 2013 Oct 22;8(10):e77851. doi: 10.1371/journal.pone.0077851 (PMC3805598; doi:10.1371/journal.pone.0077851)
Supplement: Protocol S1 — Description of procedure for liver perfusion and primary hepatocyte isolation in mice. (DOC) [file pone.0077851.s004.doc]

Protocol S1:

Euthanize mouse. Move viscera in abdominal cavity so that inferior vena cava (IVC) is exposed. Guide a suture needle/silk underneath the IVC above and below where catheter needle will enter. Puncture IVC with catheter needle and push catheter ~1cm into the IVC. Secure the catheter with the suture silks and carefully pull out the needle. Attach pump tubing to the end of the catheter. Cut portal vein and clamp off IVC above liver. Start the pump (flow rate = 5ml/min) and perfuse for 10 minutes with Liver Perfusion Medium (Life Technologies #17701-038), then 10-15 min with Liver Digest Medium (Life Technologies #17703-034) that has been pH adjusted to 7.4. Buffers should be warmed to 37C before perfusion. Stop pump when liver has expanded in size and appears somewhat translucent. Remove liver and dice in ice cold Hepatocyte Wash Medium (Life Technologies #17704-024). Draw up cell suspension and pipet through 70 µm mesh that has been placed into a sterile 50 ml conical tube. Centrifuge at 50 x g for 2 min at 4C. Pull off supernatant and add 10 ml Hepatocyte Wash Medium and 10 ml Percoll Mix to remove nonviable cells [Percoll Mix = 9 ml Percoll (GE Healthcare #17-0891-02) + 1 ml 10X PBS (Ca^2+^, Mg^2+^-free), pH adjust to 7.4]. Gently invert tube to mix, centrifuge, wash 3X with 30 ml Hepatocyte Wash Medium. Resuspend pellet in William’s E Medium (Life Technologies #12551-032) containing 10% FBS, 100U/ml penicillin, 100µg/ml streptomycin, 1nM insulin, 40ng/ml dexamethasone and 5µg/ml transferrin and count cells using trypan blue to test for viability.
