# Supplementary material for: KLF15 Is a Molecular Link between Endoplasmic Reticulum Stress and Insulin Resistance
Source: PLoS One. 2013 Oct 22;8(10):e77851. doi: 10.1371/journal.pone.0077851 (PMC3805598; doi:10.1371/journal.pone.0077851)
Supplement: Table S1 — QPCR Primer List, Related to Figures 1D, 2D, 3C and 5A. (DOC) [file pone.0077851.s005.doc]

**Table S1, QPCR Primer List, Related to Figures 1D, 2D, 3C and 5A.**

| Gene name | Forward primer | Reverse primer | GenBank Accession | PrimerBank ID |
| --- | --- | --- | --- | --- |
| *FATP/Slc27a1* | CTGGGACTTCCGTGGACCT | TCTTGCAGACGATACGCAGAA | NM_011977 | 118129963b1 |
| *FASN* | AGGTGGTGATAGCCGGTATGT | TGGGTAATCCATAGAGCCCAG | NM_007988 | 93102408b1 |
| *ApoA-IV* | ATGTGGTGTGGGATTACTTTACC | AGTGACATCCGTCTTCTGAAAC | NM_007468 | 110347472b2 |
| *KLF15* | GAGACCTTCTCGTCACCGAAA | GCTGGAGACATCGCTGTCAT | NM_023184 |  |
| *XBP-1* | AGCAGCAAGTGGTGGATTTG | CCAAGCGTGTTCTTAACTCCT | NM_013842 |  |
| *HPRT* | TGGCCATCTGCCTAGTAAAGC | GGCTCATAGTGCAAATCAAAAGTC | NM_013556 |  |
| *TBP* | ACCCTTCACCAATGACTCCTATG | TGACTGCAGCAAATCGCTTGG | NM_013684 |  |
| *B2M* | acccgcctcacattgaaatcc | cgatcccagtagacggtcttg | NM_009735 |  |
| *PGC-1α* | actgagctacccttgggatg | taaggatttcggtggtgaca | NM_008904 |  |
| *CPT1a* | GCTGCTTCCCCTCACAAGTTCC | GCTTTGGCTGCCTGTGTCAGTATGC | NM_013495 |  |
| *TNFα* | CAGGCGGTGCCTATGTCTC | CGATCACCCCGAAGTTCAGTAG | NM_013693 | 133892368c1 |
| *MCP-1* | TTAAAAACCTGGATCGGAACCAA | GCATTAGCTTCAGATTTACGGGT | NM_011333 | 6755430a1 |
